# Life is three-dimensional, and it begins with molecules

**DOI:** 10.1371/journal.pbio.2002041

**Published:** 2017-03-16

**Authors:** Philip E. Bourne

**Affiliations:** Office of the Director, National Institutes of Health, Bethesda, Maryland, United States of America

## Abstract

The iconic image of the DNA double helix embodies the central role that three-dimensional structures play in understanding biological processes, which, in turn, impact health and well-being. Here, that role is explored through the eyes of one scientist, who has been lucky enough to have over 150 talented people pass through his laboratory. Each contributed to that understanding. What follows is a small fraction of their story, with an emphasis on basic research outcomes of importance to society at large.

The three-dimensional structure of DNA, determined the year I was born, 1953 [1], has had a profound impact on science and, indeed, on my own professional life. Solving the structure of DNA laid the foundation for much of what has followed in molecular biology, evolution, genomics, the genetic basis of disease, and many more fields. The double helix is an icon for science and its progress, much like an apple with a bite out of it stands for developments in computers and that little tweeting bird for the rise of social networks.

The double helix is nature’s exquisite way of encoding biological information—alternative arrangements of phosphates and sugars covalently bound in a right-handed helix. Each unit of phosphate and a five-carbon sugar (deoxyribose) form a nucleotide. Add one of four nitrogenous bases, adenine (A), cytosine (C), guanine (G), and thymine (T), and you can build, as Watson and Crick did in 1953, two complementary strands that encode the genetic blueprint of life as a series of four letters. For humans, that’s 3,000,000,000 letters in all, arranged into 23 chromosomes. The DNA molecule personifies the three-dimensional nature of life, and it is that feature, which has intrigued me my whole career.

The fun begins when you consider that three-dimensional chemical structures can be determined by experimentally applying the laws of physics and computational tools. The Braggs, father and son, showed in 1913 [2] that crystalline solids produced patterns of diffracted X-rays. The patterns flow from the dimensions of the repeating crystal lattice. The discovery that the intensity of the resultant diffraction spots is a function of the position of the atoms—more specifically, their electron clouds—affirmed the atomic nature of matter but also birthed the field of X-ray crystallography [3].

Many years later, in 1975, I began my graduate career in Adelaide, Australia, where the Braggs’ pioneering work had been carried out. Using the technique of X-ray crystallography, I determined the structures of components of DNA bound to small molecules, notably, cis-platinum drugs and variants thereof. The goal was to determine the mode of binding and ascertain whether more powerful and hopefully less toxic drugs could be found. Cisplatin, the simplest of this class of compounds, was approved by the Food and Drug Administration in 1978 and is still in use today [4]. Cisplatin attacks tumors by linking the DNA strands to disrupt cell replication. Unfortunately, it also affects normal cells, leading to undesirable side effects. Notwithstanding, it establishes the role that three-dimensional structures play in the early stages of the drug discovery process. That some aspects of a drug’s effectiveness come down to subtle interactions of individual atoms is remarkable, and we will revisit this below after 30 years of additional insight.

Writing and running computer programs that defined the positions of atoms in matter was so cool, I was hooked. Without the benefit of the computer graphics displays that exist today, atomic positions were drawn out of perspex sheets as contours: just as in geographic relief maps, the close proximity of contours represented a steep hill and peak, but here, they identified peaks of electron density—the position of atoms. The thrill of mapping out atomic structures that no one had seen before remains with me to this day.

As techniques, computers, and instrumentation advanced, so did the size and number of determined molecular structures. As a postdoctoral fellow in Sheffield, United Kingdom, in the late 1970s, I turned my hand from a few dozen atoms to thousands. Working on the iron-storage protein, ferritin, we determined that ferritin was composed of 24 individual protein chains arranged in perfect symmetry to form a spherical molecule capable of storing 4,500 iron ions [5]. There were only about 80 protein structures at the time, several of which had led to Nobel prizes because, like ferritin, they helped illuminate biological function and, indeed, malfunction. The ferritin structure increased our understanding of iron metabolism and directly associated disease states such as neuroferritinopathy, a neurodegenerative disorder associated with a mutation in the ferritin light chain. A single letter change in the DNA of the ferritin gene leads to a significant change in the resultant protein structure. A change profound enough to impact iron uptake. One result of iron and ferritin deposition in the brain is a movement disorder, which can now be diagnosed with genetic testing. Measuring serum ferritin levels is now a standard diagnostic test for a variety of diseases that involve iron deficiency.

A few years later at Columbia University in New York, just as workstations and personal computers were emerging, my engineering side took over, and I got interested in building both hardware and software using these new tools. There, I would have likely remained if not for a bold new initiative—the human genome project. Until then, those of us who used computers to solve biological problems were, to put it politely, outliers. Suddenly, we were an integral part of the discovery process—vital contributors to both the assembly of the genome and the management of the large amount of digital data resulting from the project. It was 1995, we were called bioinformaticians, and I could see what was coming—biomedicine as a computational science.

Combining my acquired computational skills with my structural biology skills, I started asking questions utilizing the complete corpus of structural information—by then, at 3,800 structures and growing fast. To analyze the data, we needed databases for fast and organized access and a consistent description of the data for comparative analysis. Work in this area ultimately led to a group of us maintaining the Protein Data Bank (PDB), a worldwide repository for biological structure information [6]. It was immensely rewarding to help provide a resource that was used by many thousands of researchers each month. Ironically, database developers, biocurators, and others responsible for community data sharing remain the unsung heroes of biology, as they remain undervalued in the academic system. Academia has yet to fully appreciate the digital era, but that is another story. This story is about a joyous, winding career path and the science that, hopefully, would eventually be valuable to the public. That research went in many directions but much was driven by what could be done with the PDB. Here, I will focus on two quite different aspects of that research—evolution and drug discovery—and what our laboratory contributed.

Perhaps my favorite fact in all biology is that proteins, transcribed and translated from DNA, consist of 20 amino acids strung together in a polypeptide chain. Assuming an average chain length of 300 amino acids, this leads to 20^300^ possibilities, more than all the atoms in the universe. Yet, to our knowledge, at least according to RefSeq [7], a database of protein reference sequences, we have only discovered 79 million proteins to date. There are undoubtedly many to be discovered, but nevertheless, it will be a very small number compared to what is possible. Even more astounding is that these 79 million one-dimensional strings fold into approximately 1,000 unique protein three-dimensional folds [8]. Amazing as it is, all life is composed of 1,000 three-dimensional jigsaw pieces put together in a multitude of ways—nature’s reductionism. How can we use this fact to study evolution? It follows that with so few folds, the invention of a new fold during evolution, or the frequency with which a fold is used, is a major event. Studying these events, previously done with DNA and protein sequences alone, we—and others—have determined how the changing geochemistry of the Earth, as reflected in new protein folds, and how the changing usage of those folds impacted the development of life [9]. Looking forward, not only do we have a tool to understand the impact of environment on life, but we can also engineer proteins that nature has yet to discover or discovered then discarded. This research has many implications, for example, understanding climate change, increasing food production, and increasing energy production from biofuels.

Consider a completely different aspect of life in three dimensions—drug discovery. A simple-minded view of drug action is to find a small molecule—like cisplatin, which we met earlier—and have it bind to a protein, DNA, or RNA target, thereby modifying its action and having a deleterious effect on a disease condition. If only it were that simple. Drug side effects arise for a number of reasons, an important one being that drugs do not bind to a single target but to multiple targets with varying affinity. What we as drug takers are actually experiencing is a collective effect—an effect on the complete system. To add to the complexity, we each experience a different collective effect, given our specific genetic, physiological, and environmental states.

Understanding this complex human system and designing drugs that lead to improved health outcomes is referred to as systems pharmacology [10], and we are making progress. It begins with an understanding of the three-dimensional nature of how drugs bind to their targets at scale. The three-dimensional chemical space occupied by drugs and biological targets is huge, but the 80 protein structures we had in 1980 has now grown to approximately 125,000, and it is possible to algorithmically compare the similarity of binding pockets across this complete set of proteins. We increasingly know what other molecules these proteins interact with, what biochemical pathways they can be found in, and much more. Putting this together, we can begin to make computational predictions about the effectiveness of a given compound, which can inform experimental testing. The promise is to accelerate the drug discovery process and to facilitate personalized medicine, where the dose and type of drug match our specific genetic and physical profile.

This brief tour of one person's research career spans 40 years, starting with simple three-dimensional molecules and ending with the beginnings of understanding human health as a computable system. What a privilege it has been to contribute a tiny part to this progress. While we hear of the big breakthroughs in science, it is important to remember that those breakthroughs, and the many smaller findings, are the accumulated efforts of many scientists who openly share their work. Collectively, this positively impacts all aspects of our lives. Want proof? During my days on Earth, life expectancy worldwide has risen from 47 to 73 years of age [11]. If that is not success, I don't know what is.

**Fig 1:**
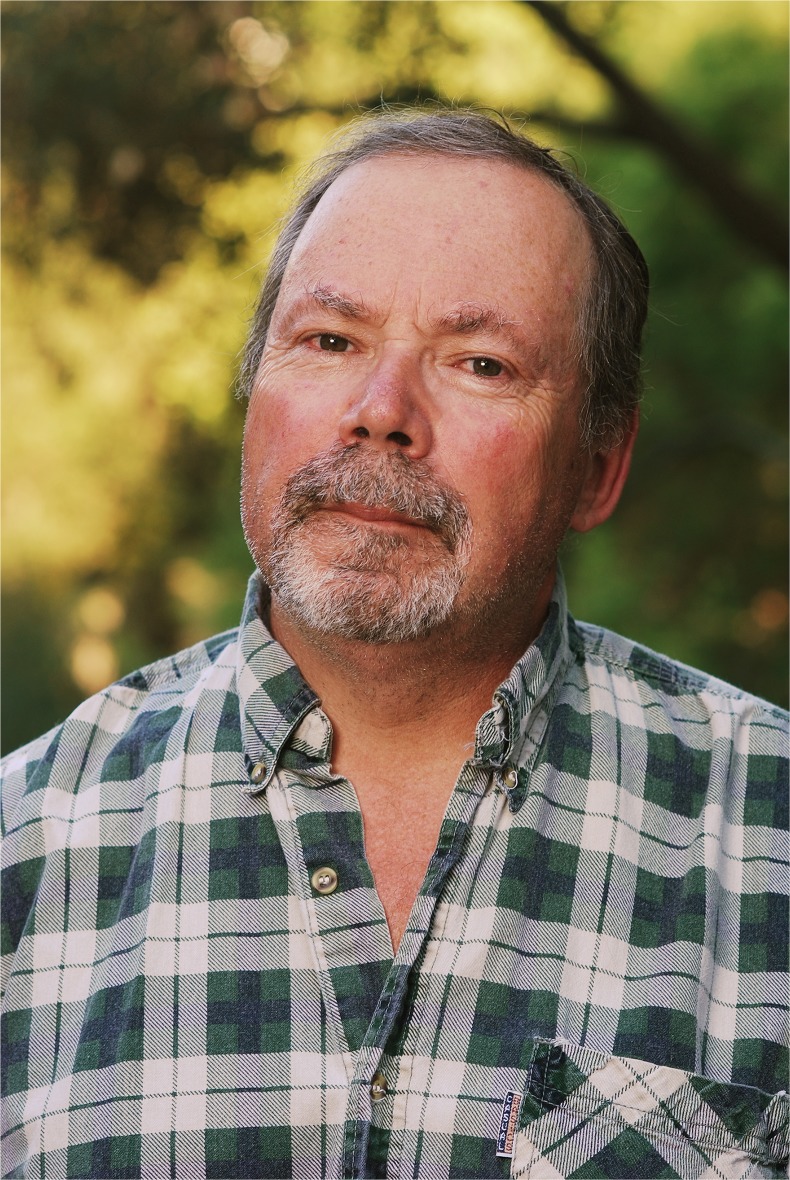
Philip E. Bourne.
